# Self-Assembling Peptides as Extracellular Matrix Mimics to Influence Stem Cell's Fate

**DOI:** 10.3389/fchem.2019.00172

**Published:** 2019-03-27

**Authors:** Katharina S. Hellmund, Beate Koksch

**Affiliations:** Department of Biology, Chemistry, and Pharmacy, Institute of Chemistry and Biochemistry, Freie Universität Berlin, Berlin, Germany

**Keywords:** extracellular matrix, stem cells, peptide-based biomaterials, self-assembling peptides, peptide hydrogels, stem cell differentiation, stem cell fate

## Abstract

Interest in biologically active materials that can be used as cell culture substrates for medicinal applications has increased dramatically over the last decade. The design and development of biomaterials mimicking the natural environment of different cell types, the so-called extracellular matrix (ECM), is the focus of research in this field. The ECM exists as an ensemble of several adhesion proteins with different functionalities that can be presented to the embedded cells. These functionalities regulate numerous cellular processes. Therefore, different approaches and strategies using peptide- and protein-based biopolymers have been investigated to support the proliferation, differentiation, and self-renewal of stem cells, in the context of regenerative medicine. This minireview summarizes recent developments in this area, with a focus on peptide-based biomaterials used as stem cell culture substrates.

## Introduction

Developing cell culture materials is a challenging task for the chemistry community, but its successful realization would allow cellular behavior to be directly influenced in order to optimize their applications in regenerative medicine. By tuning the properties of such materials, i.e., stiffness or the presence of chemical inducers like recognition motifs, it is possible to tailor the stem cell's microenvironment to give information regarding proliferation and differentiation. In nature, stem cells are arranged in a defined microenvironment, referred to as stem cell niche, which regulates the stem cell's behavior based on input from all of the components that comprise it (Jones and Wagers, 2008; Kühl and Kühl, 2012). The stem cell niche theory was developed by Schofield (1978) and describes how stem cells are supported or influenced by the defined microenvironment, by means of physical interactions between the stem cells and other cells, the secretion of signal molecules, or the presence of molecules on the surface of other cells, like integrins (Fuchs et al., 2004; Li and Xie, 2005; Jones and Wagers, 2008; Kühl and Kühl, 2012).

The extracellular matrix (ECM) is the major and most important part of the stem cell niche. Numerous studies have shown that ECMs influence stem cell's fate (Guilak et al., 2009). The composition of natural ECM can differ and depends on its tissue of origin. It consists of different adhesion proteins like collagen, fibronectin, and laminin, to which the stem cells are exposed (Hynes, 2009; Kühl and Kühl, 2012; Watt and Huck, 2013). These adhesion proteins bind to integrins on the cell surface via cell-binding epitopes. Cell-binding epitopes are small peptide sequences derived from adhesion proteins, namely, RGD from collagen, RGDS from fibronectin and IKVAV and YIGSR from laminin (Lampe and Heilshorn, 2012). The stem cell senses the environment in which it is embedded and is able to modify its form and function accordingly; this is its main mechanism of proliferation and differentiation. Understanding this mechanism in detail is of paramount importance to the development of new biomaterials for stem cell biology (Lutolf et al., 2009).

Stem cells have specific characteristics that differ from those of other cells in the body. In particular, they are able to differentiate into individual cell-types (Ding and Schultz, 2004; Griffin et al., 2015) and contribute to the regeneration of tissues (Kühl and Kühl, 2012; Griffin et al., 2015). Additionally, the unlimited self-regeneration by asymmetric cell division into a specialized and an unspecialized daughter cell constitutes one of the main characteristics of stem cells (Watt and Hogan, 2000; Knoblich, 2008). Two different types are known: embryonic stem cells (ESCs) and adult stem cells (ASCs) (Kühl and Kühl, 2012; Griffin et al., 2015). ESCs are derived from the inner cell mass of the blastocyst (Ding and Schultz, 2004; Kühl and Kühl, 2012) and have a pluripotent differentiation potential (Odorico et al., 2001; Kühl and Kühl, 2012). Induced pluripotent stem cells (iPSCs) are a nearly inexhaustible source of pluripotent stem cells. They are directly reprogrammed from fibroblasts by introduction of four growth factors Oct3/4, Sox2, c-Myc, and Klf4. iPSCs possess the properties similar to ESCs (Takahashi and Yamanaka, 2006) and are therefore an auspicious cell species to study the differentiation potential of artificial cell culture substrates for differentiation of pluripotent stem cell lines. ASCs are unspecialized, multipotent cells derived from adult tissues and can differentiate into restricted types of specialized cells (Ding and Schultz, 2004; Griffin et al., 2015). The most common type of ASCs are mesenchymal stem cells (MSCs) ASCs require a defined microenvironment in an adult organism to maintain their stem cell properties.

Over the last decade, the importance of identifying and exploring ECMs and their niches has been increasingly recognized (Morrison and Spradling, 2008; Walker et al., 2009). Peptides and peptide derivatives are excellent candidates for the development of 2D and 3D cell-culture materials, thanks to their biodegradability and biocompatibility, and make it possible to tailor biomaterials for different applications in tissue engineering and regenerative medicine (Lampe and Heilshorn, 2012; Wu et al., 2012). Specific types of peptide-based materials possess specific advantages: whereas 2D scaffolds present highly sensitive ligands bound to an inert substrate, elasticity and stiffness are key in obtaining differentiation signals for stem cells embedded in 3D scaffolds. The properties of each peptide-based material can be adjusted by the primary structure, and they are generally easy to procure by means of standard peptide synthesis protocols (Jung et al., 2010). Peptide structures, folding processes and stability are also for the most part well-established in the literature (Lakshmanan et al., 2012; Wu et al., 2012).

## Parameters of a Synthetic Extracellular Matrix

Stem cells are sensitive toward the physical parameters of the surrounding microenvironment and are able to adapt their differentiation lineage according to the topology, polarity or the elasticity of the surface (Trappmann et al., 2012; Murphy et al., 2014).

**Figure 1:**
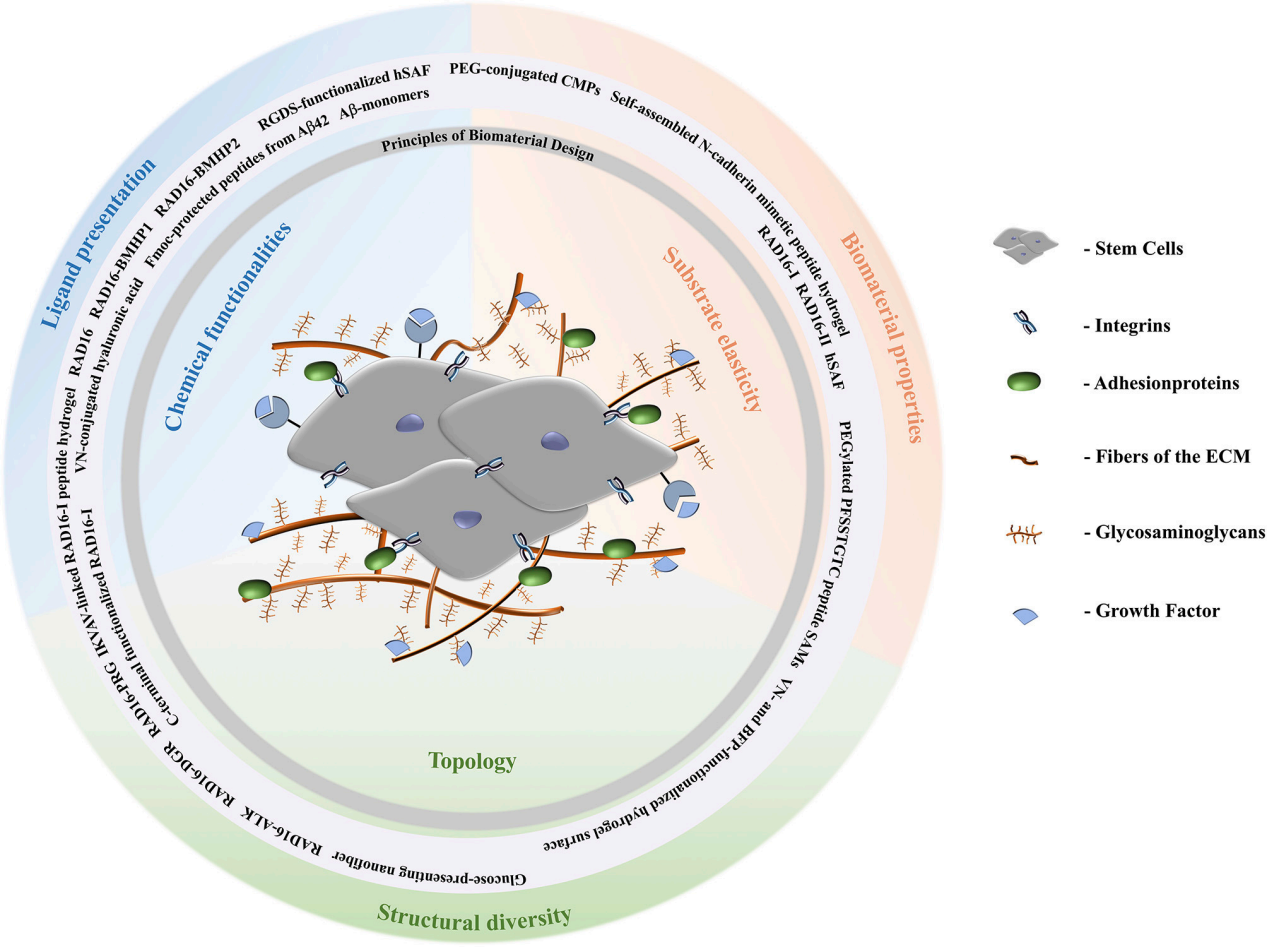
Schematic representation of the extracellular matrix of stem cells. Stem cells are surrounded by fibers and adhesion proteins which recruit integrins. Their fate is directed by aspects of the ECM like stiffness, cell-cell interactions, and composition with respect to solubility factors, adhesionproteins, and glycosaminoglycans. Topological signals can be epitopes presented by the latter to direct cell behavior. According to the biomaterial design principles discussed, peptide materials can be designed to comply with the requirements of the natural ECM. A stiff ECM leads to differentiation toward stiff tissues, i.e., osteogenesis. Depending on the specific lineage of stiffness and elastic moduli, for example, MSCs can differentiate tissues according to stiffness of tissues the cells are specializing in. Brain tissue has elastic moduli that range from 0.1 to 1 kPa and can entrap neurocytes (Lv et al., 2015); elastic moduli of pancreatic tissue are about 1.2 kPa; cartilage tissue has a typical elastic modulus of 3 kPa and entraps chondrocytes; muscle tissues has elastic moduli between 8 and 17 kPa and entraps myoblasts; and the strongest is osteoblast entrapping bone tissue with elastic moduli from 25 to 40 kPa (Aurand et al., 2012; Lv et al., 2015).

Non-peptidic topological cues influencing stem cells include surfaces presenting different chemical functionalities; for example, glass silane modified substrates with diverse functional groups (methyl-, amino, silane-, hydroxy-, and carboxy-groups) on their surfaces were established to culture hMSCs (Curran et al., 2006). Methyl-substituted surfaces lead to maintenance of the hMSC phenotype, amino-surfaces promote and maintain osteogenesis, and hydroxyl- and carboxyl-groups promote and maintain chondrogenesis (Curran et al., 2005, 2006). Stiffness is one of the main parameters for directing the fate of a stem cell, and it is expressed by an elastic modulus. Stem cells are mechanically sensitive, thus their fate is directed by the stiffness of the environment into which it is embedded (see Figure 1). Examples of stiffness cues include the development of biomaterials for directed stem cell differentiation in 2D and 3D cell cultures. For example, polyethylene glycol silica nanocomposite gels containing RGD-peptides and exhibiting different degrees of stiffness were investigated regarding hMSC differentiation. Stiffer gels showed higher expression levels of Runx2, an early bone differentiation transcription factor, compared to gels having low or intermediate stiffness (Pek et al., 2010).

## Cell Attachment and Proliferation on Peptide-based Biomaterials

A prominent example of conjugation of adhesion proteins with different biologically relevant polymers was shown by Zhang et al. (2016), who functionalized hyaluronic acid (HA) with methacrylate to achieve photo-crosslinking on glass-slides and conjugation of a synthetic peptide derived from vitronectin (VN) to the glycosaminoglycan (Zhang et al., 2016). The growth rate of hiPSCs on these VN-MeHA surfaces was compared to that on Madrigal, a mixture of ECM proteins, and similar results were obtained (Zhang et al., 2016).

Another example of a 3D nanofibrous scaffold supporting cell differentiation is the RAD16 peptide family (Zhang et al., 2005; Wu et al., 2012) These two ionic peptides, named RAD16-I and RAD16-II, are able to form stable β-sheets in water and self-assemble spontaneously into nanofiber scaffolds, which could mimic the ECM of tissue cells (Yanlian et al., 2009; Wu et al., 2012). Furthermore, three different self-assembling peptide hydrogels were developed to mimic a 3D cell environment for human adipose stem cells by functionalization of RAD16-I with three biologically active peptide motifs (see Table 1) at its C-terminus (Liu et al., 2013). The functionalized RAD16-I peptide-based hydrogels were used for cell culture and showed that the peptide motifs increased the viability of human adipose stem cells compared to RAD16-I. The largest number of human adipose stem cells showing highest biological activities including migration, proliferation and growth factor-secretion were detected in case of RAD/PRGD. These kinds of biomaterials follow simple design principles and are easy to connect with biologically relevant peptide epitopes to tune bioavailability.

**Table 1:** Differentiation potential of 2D and 3D peptide-based biomaterials, differentiation features: 1, chemical group; 2, substrate elasticity; 3, topology.

**Cell behavior**	**Cell type**	**Substrate description**	**Feature**	**References**
Cell attachment and proliferation	hiPSC	VN (Ac-KGGPQVTRGDVFTMP)-conjugated hyaluronic acid	1	Zhang et al., 2016
	hASC	C-terminal functionalized RAD16-I: Bone Marrow Homing Peptide 1 (BMHP1, SKPPGTSS, RAD/SKP), heparin binding motif (FHRRIKA, RAD/FHR) and a PRGD-peptide motif (PRGDSGYRGDS, RAD/PRGD)	1	Liu et al., 2013
	hMSC	Fmoc-protected peptides from Aβ42	1, 3	Jacob et al., 2015
Neurogenesis inducing materials	hMSC	Fmoc-protected peptides from Aβ42	1, 3	Jacob et al., 2015
	ratNSC	Aβ-monomers	1	Collins et al., 2015
	ratNSC	IKVAV-linked RAD16-I peptide hydrogel	1, 2, 3	Cheng et al., 2013
	murine NSC	RAD16, RAD16-BMHP1, RAD16-BMHP2 (PFSSTKT)	1, 3	Gelain et al., 2006
	rat PC12	RAD16-I, RAD16-II	3	Holmes et al., 2000; Wu et al., 2012
	rat PC12, murine NSC	hSAF, RGDS-functionalized hSAF	1, 2	Banwell et al., 2009; Mehrban et al., 2015
Chondrogenesis inducing materials	MSC, Chondrocytes	PEG-conjugated CMPs	3	Lee et al., 2008; Deans and Elisseeff, 2010
	hMSC	Self-assembled N-cadherin mimetic peptide hydrogel	1, 2	Li et al., 2017
	ratMSC	Glucose-presenting peptide nanofiber	1	Yasa et al., 2017
Osteogenesis inducing materials	ratMSC	Glucose-presenting peptide nanofiber	1	Yasa et al., 2017
	hiPSC	VN- and BFP- functionalized hydrogel surfaces	1	Deng et al., 2018
	MSC	PEGylated PFSSTGTC peptide SAMs	1, 3	Han et al., 2015
	hMSC	Covalently grafted KRGDSPC modified silica-nanoribbons	1, 3	Das et al., 2013
	ratMSC	N-terminal palmitic acid modified RGDEAAAGGG	1, 2, 3	Hosseinkhani et al., 2006a,b
	MC3T3	RAD16, RAD16-ALKRQGRTLYGF (ALK), RAD16-DGRGDSVAYG (DGR), RAD16-PRGDSGYRGDS (PRG)	1, 3	Horii et al., 2007
	Primary rat osteoblasts, putative rat liver progenitor cells	RAD16-I coated H-PHP	1, 3	Semino et al., 2003; Bokhari et al., 2005

Beside nanofibrous scaffolds, amyloids represent promising peptide species as substrates to control stem cell behavior. Amyloids are highly ordered peptide aggregates. Their formation from soluble proteins during the life-time of an organism is commonly associated with degenerative diseases including Alzheimer's and Parkinson's disease. It may seem unexpected to use motifs that are responsible for degenerative diseases as substrates for applications in regenerative medicine, but recent studies have shown that this is possible (Masliah et al., 2001; Hardy, 2002; Lashuel et al., 2002; Chiti and Dobson, 2006; Winner et al., 2011; Jacob et al., 2015). Amyloid folding can be tuned based on the amino acid composition (Mankar et al., 2011). Amyloid structures exist predominantly as β-sheets, which are characteristically arranged in a highly repetitive cross-β structure, which forms stable, long, straight, and unbranched amyloid-fibrils consisting of individual subunits, named protofilaments (Goldsbury et al., 1999; Serpell et al., 2000; Nelson and Eisenberg, 2006; Rambaran and Serpell, 2008). Jacob et al. developed a series of peptides to form amyloid nanofibril based hydrogels for 2D and 3D stem cell culture and differentiation (Jacob et al., 2015). These amyloid nanofibrils, consisting of self-assembling Fmoc-protected peptides derived from β-sheet prone C-terminal Aβ42 are non-toxic, thermo-reversible and thixotropic. By varying the peptide and salt concentration, the stiffness of the resulting amyloid gels can be modulated. Most of them are supporting cell attachment, proliferation and influence the stem cell fate. The softest gel supports neuronal differentiation pathways for hMSCs. In summary, stem cell proliferation and adhesion can be supported by fibrous structures that provide a 3D environment which approximates the natural ECM. Additional approaches also use short self-assembling Fmoc-peptides as scaffolds, e.g., for the delivery of growth factors (Rodriguez et al., 2013, 2018; Bruggeman et al., 2016) or enzymatic self-assembly control (Williams et al., 2010). Furthermore, conjugation with short biologically relevant peptides enhances biocompatibility and differentiation activity.

## Neurogenesis Inducing Biomaterials

Besides the described physical hydrogel support, it is well-known that β-amyloid peptide (Aβ) and prion protein (PrP), the Aβ-oligomer receptor, both influence neurogenesis. Studies by Collins *et al*. showed that neural stem cells (NSCs) were influenced by this interplay via different Aβ-pathway activation in presence or absence of PrP. The proliferation signals of NSCs were inhibited by Aβ-PrP signaling (Collins et al., 2015). Short regions of the amyloid precursor protein, especially Aβ42, could be suitable for the development of amyloid-based materials for NSCs cell culture and differentiation.

In contrast to β-sheet based amyloids, certain coiled coil forming peptides can adopt higher ordered structures that are α-helical in nature. Coiled-coil motifs are common among natural proteins, in fact, sequence analysis has shown that nearly 2–3% of natural proteins contain coiled-coils (Wolf et al., 1997; Burkhard et al., 2001). The coiled-coil consists of two to seven α-helical strands that form a left-handed superhelical twist (Woolfson, 2005; Falenski et al., 2010). Its amino acid sequence is characterized by a seven residue periodicity, called a heptad repeat and enables the rational design by modification, for example with carbohydrate or peptide ligands (Zacco et al., 2015a,b).

Based on this design, several groups have developed peptide-based materials for cell culture applications. A self-assembling α-helical peptide-based hydrogel was shown to support the growth and differentiation of rat adrenal pheochromocytoma cells. The developed hydrogelating self-assembling fibers (hSAFs) are able to form networks of α-helical fibrils and build hydrogels, which entrap more than 99% of water by weight. The design of a self-assembling fiber (SAF) based on an α-helical coiled-coil peptide was previously described (Ryadnov and Woolfson, 2003; Papapostolou et al., 2007; Banwell et al., 2009). The resulting hSAFs were applied as suitable and defined microenvironments for cell growth and differentiation. A RGDS-functionalized hSAF demonstrated improved cellular attachment and differentiation compared to undecorated gels for NSC culture (Mehrban et al., 2015). NSC attachment to the developed hSAFs increases upon addition of the cell-adhesion motif RGDS. It was also shown that the NSCs form large neurospheres inside the functionalized gels and then mature into neurons. Thus, it was demonstrated that these α-helical peptide-based hydrogels provide an appropriate extracellular environment for NSCs and represent a starting point for the development of materials that support neural tissue engineering.

Additionally, nanofibrous structures, like RAD16, were shown to support gene expressions of neural stem cell differentiation by conjugation with BMHP1 and Bone Marrow Homing Peptide 2 (BMHP2) (Gelain et al., 2006). The conjugation of RAD16-I with IKVAV was realized with the aim of creating a self-assembling IKVAV-linked peptide hydrogel as a 3D scaffold for enhanced differentiation of neural stem cells and is a potential application for the regeneration of injured brain tissue (Cheng et al., 2013). The IKVAV motif is known to promote neurite outgrowth and enhances neuronal differentiation and neurite proliferation (Yamada et al., 2002). Similarly to RAD16-I, RAD16-II showed to be a suitable substrate for neurite outgrowth (Holmes et al., 2000; Wu et al., 2012). The conjugation of different peptide motifs to these peptide hydrogel scaffolds lead to an enhancement of neuronal differentiation behavior of different stem cell lines.

## Chondrogenesis Inducing Biomaterials

Mimicking the structure of adhesion proteins like collagen is one approach to creating chondrogenesis inducing materials (Zhang et al., 2012). Collagen has a triple-helix structure (Brodsky and Ramshaw, 1997; Brodsky and Persikov, 2005; Wess, 2005) that can be mimicked by synthetic peptide-amphiphiles (Cen et al., 2008; Zhang et al., 2012) and coiled-coil peptides (Hennessy et al., 2009). Important examples include collagen mimetic peptides (CMPs), which are able to adopt the tertiary structure of natural collagen (Lee et al., 2006, 2008; Deans and Elisseeff, 2010). Conjugated with PEG, the CMP substrates retain their structure to help encapsulate collagen, which maintains chondrocytes (Lee et al., 2006; Deans and Elisseeff, 2010) and thus, serves as suitable substrate to enable MSCs chondrogenesis (Lee et al., 2008; Deans and Elisseeff, 2010). Also, a self-assembled N-cadherin mimetic peptide hydrogel was found to promote chondrogenic differentiation of hMSC (Li et al., 2017).

Another approach investigates the effect of glucose, carboxylate, and sulfonate attached to a peptide nanofiber on the differentiation behavior of rat MSCs (Yasa et al., 2017). It was shown that the presence of a glucose moiety on the peptide nanofiber leads to osteo/chondrogenic differentiation of the tested cells. Chondrogenesis inducing peptidic biomaterials have to combine the structural and morphological features of cartilage tissue. Glycosaminoglycans play an important role in achieving the stiffness of natural ECM, and conjugation of simple functional groups to these substrates is a promising proof-of-concept to represent the complexity of natural ECMs.

## Osteogenesis Inducing Biomaterials

Bone tissue has the greatest stiffness of all types, but stem cells exist in the bone marrow niche, which is variable stiff and it is not clear how their fate will be directed after leaving their niche (Ivanovska et al., 2015). In addition to stiffness, substrates that enhance osteogenesis have to provide topological cues to induce osteogenic signals.

Self-assembled monolayers (SAMs) are highly organized substrates, which are adsorbed onto gold surfaces and suitable for development of 2D-cell culture materials due to tunable surface properties to regulate cell-substrate interaction via surface modifications (Hudalla and Murphy, 2009; Griffin et al., 2015; Kehr et al., 2015). To this end self-assembling peptides are optimal substrates; for example, a SAM consisting of PEGylated PFSSTKTC peptide modified on quartz substrate was shown to direct osteogenic differentiation of MSCs (Han et al., 2015).

The modification of hydrogels with biologically relevant peptide epitopes, derived from adhesion proteins of the ECM was shown to support long-term maintenance and induce osteogenic differentiation of hiPSCs. The VN and BFP (Bone Forming Peptide) concentration on the hydrogel surface was varied and different degrees of differentiation of hiPSCs were achieved (Deng et al., 2018).

Modification and covalent grafting of silica-nanoribbons with KRGDSPC peptide onto activated glass substrates highlights the influence of shape and topology of a synthetic ECM on cell fate. It was shown, that the studied helical nanoribbons induced differentiation into osteoblast lineage (Das et al., 2013).

C-terminal extension of RAD16 with different biologically relevant epitopes are shown to promote osteogenic differentiation of osteoblast precursor cells (Horii et al., 2007). Further developments investigated the design of peptide-amphiphiles including the recognition motif RGD (see Table 1) and N-terminal palmitic acid. This conjugate builts a 3D-network by mixing an aqueous solution of peptide-amphiphile with MSCs and influences the stem cells' attachment, proliferation, and differentiation to osteogenic lineage (Hosseinkhani et al., 2006a,b). Combination of PolyHIPE polymer (PHP) with RAD16-I produced a nanoscale environment and spontaneously self-assembled into highly hydrated nanofibers able to trap volume contents of 99.5% water and to envelope cells in a 3D environment (Semino et al., 2003; Bokhari et al., 2005). A significant increase of cell-number in RAD16-I coated H-PHP constructs was observed compared to H-PHP alone and osteoblast differentiation was observed (Bokhari et al., 2005). Several approaches dealing with osteoinductive materials are focusing on signals sent by molecules covalently grafted onto inert surfaces or presented in a 3D nanofiber network.

## Conclusion

With the help of 20 canonical amino acids nature builds a repertoire of structural motifs to determine stem cell fate. This repertoire can be extended via the use of synthetic amino acids and in creating biologically relevant recognition and signal motifs. The resulting structural motifs can build stable, flexible and regenerative assemblies to form artificial ECMs. The peptide-based materials presented in this review comply with the requirements of an artificial matrix for mimicking the natural environment of stem cells. It is important that such peptides and their resulting assemblies be stable enough to remain in stem cell culture medium without precipitation due to undesired further aggregation or degradation. Peptides have been successfully used to develop scaffolds of variable stiffness for stem cell culture and differentiation and have proven their potential for use as ECM mimetics and as potential stem cell culture substrates. The advantageous properties of these biopolymers include their ease of design and synthesis and their ability to form chemically well-defined higher order assemblies, which makes them ideal candidates for both 2D and 3D tissue engineering applications. In this context, coiled-coil peptides have proven as highly suitable scaffolds for cell culture applications due to their predictable self-assembly properties, which also allow multivalent ligand presentation (Zacco et al., 2015a). However, to date, very few examples of 2D and 3D coiled-coil based scaffolds that influence stem cell behavior exist. Another limitation is our current understanding of stem cell niches and their differentiation mechanisms, which is not sufficient yet for either the rational design of ECM-materials or the prediction of the fate of the cell; more research is needed in this area.

## Author Contributions

All authors listed have made a substantial, direct and intellectual contribution to the work, and approved it for publication.

### Conflict of Interest Statement

The authors declare that the research was conducted in the absence of any commercial or financial relationships that could be construed as a potential conflict of interest.
